# Pulmonary scarring and its relation to primary lung cancer

**DOI:** 10.7196/AJTCCM.2020.v26i1.050

**Published:** 2020-03-19

**Authors:** S Brett, E M Irusen, C F N Koegelenberg

**Affiliations:** Division of Pulmonology, Department of Medicine, Faculty of Medicine and Health Sciences, Stellenbosch University and Tygerberg Academic Hospital, Cape Town, South Africa

## Abstract

**Background:**

Lung scar carcinoma, so called ‘scarcinoma’, is a perceived entity that was originally described by Friedrich in 1939, in which a carcinoma originates from peripheral scarring of lung tissue. In a recent pilot study, there was a strong association between the geographic location of lung cancer and the presence of scarring of the lung.

**Objectives:**

To investigate this relationship in the largest cohort to date.

**Methods:**

We reviewed all radiological images of patients (N=917) with confirmed lung cancer from 2013 - 2017 and included all who had at least a staging computed tomography (CT) of the chest and a tissue diagnosis of primary lung cancer. Two pulmonary specialists categorised all patients as no pulmonary scarring, scarring in the same lobe, scarring in the ipsilateral lung, but not lobe, scarring in the contralateral lung and diffuse scarring both lungs.

**Results:**

Almost 1 in 3 patients had pulmonary scarring. In patients with lung cancer, if scarring was present, the pulmonary scarring was more likely to be found in the same lobe as the cancer compared with any other lobe, including the same lung (p<0.0001).

**Conclusion:**

Pulmonary scarring was common, and there was a strong association between the geographical location of scarring and primary lung cancer in those with scarring.

## Background

In 2013, lung cancer was the third-most common cancer in men and sixth-most common cancer in women in South Africa (SA).^[1]^ It is also the leading cause of cancer-related deaths in SA.^[2]^ Tobacco use is the traditional risk factor for development of lung carcinoma, but elevated risk is seen in conditions predisposing to inflammatory states (e.g. tuberculosis (TB) and pneumonia).^[3,4]^ A shared feature of these diseases is that of pulmonary fibrosis (scarring), and this has long been thought to be a risk factor for the development of lung carcinoma.^[5]^

Lung scar carcinoma, so-called ‘scarcinoma’, is a concept that was originally described by Friedrich in 1939 in which a malignancy originates from peripheral scarring of lung tissue.^[6]^ It is defined as any peripherally located tumour <3 cm occurring in intimate relation to scar tissue, with no evidence of bronchial origin. It is usually found in males in the upper lobes of the lungs. Histologically this is typically an adenocarcinoma.^[7–11]^ Pulmonary fibrosis (i.e. scarring) is a sequela of cellular inflammation and wound repair characterised by the deposition of connective tissue. Pulmonary scarring can result from lung diseases caused by a variety of occupational exposures, such as to asbestos and silica, and inflammatory or infectious lung conditions, such as tuberculosis and other forms of pneumonia. It also occurs for unknown reasons (e.g. idiopathic pulmonary fibrosis).^[5]^

In a pilot study conducted at Tygerberg Hospital in Cape Town, SA, one in five patients with primary lung carcinoma had scarring present on staging computed tomography (CT) scans.^[12]^ In another study by Yu *et al*.,^[5]^ it was found using the Prostate, Lung, Colorectal and Ovarian database that 15% of patients with lung carcinoma had lung scarring at baseline. Apart from these two studies, both of which were small, there are limited data on scar carcinoma as a separate entity or as a risk factor for lung cancer. The close anatomical location of scarring to a primary lung tumour, possible genetic variations and their behaviour^[13]^ would favour scar carcinoma as a separate entity.

In 2007, the Burden of Obstructive Lung Disease study^[14]^ showed that Cape Town had a high smoking prevalence of 56.9% in males and 40% in females, and one in five patients had a history of pulmonary TB. Our aim was to investigate the relationship between scarring and lung cancer in the largest cohort to date, specifically in Cape Town, with its high prevalence of smoking and large tuberculosis burden.

## Methods

We retrospectively reviewed an existing lung cancer registry and included all radiological images of patients >18 years of age with confirmed lung cancer at Tygerberg Hospital in Cape Town, SA, from 2013 to 2017. We included all patients who had a least a contrasted staging CT scan of the chest and a histological diagnosis of primary lung cancer. The radiological images included chest radiographs and contrasted CT scans of the chest.

Routine demographic and clinical data were recorded. Smoking history was recorded where available, using the existing database as well as patient folders found in Tygerberg Hospital’s electronic filing system. The patient’s functional status was defined based on the Eastern Cooperative Oncology Group (ECOG) Performance Status. Clinical staging was based on the 8th edition of tumournode-metastasis for lung cancer.^[15]^

CT scans were reviewed by two pulmonary specialists (not blinded to the other’s opinion) for evidence and location of pulmonary fibrosis, be it localised or diffuse. Patients who had scarring deemed to be due to the primary cancer or from metastatic lesions (i.e. a desmoplastic reaction) by both investigators were excluded. Where there was doubt about this the patient was not labelled as having pulmonary scarring if it occurred only in that lobe.

**CT features of fibrosis were reported as scarring. This included the presence of one or more of the following features:**

- lung architectural distortion, which included cavities and fibrocystic changes
- honeycombing
- bronchiectasis
- reticulation (both focal or diffuse)

**The patients were then categorised as having:**

- no pulmonary scarring
- any scarring in the same lobe as the primary tumour
- any scarring in the ipsilateral lung, but not in the lobe of the primary tumour
- any scarring in the contralateral lung
- diffuse scarring in both lungs (e.g. idiopathic pulmonary fibrosis)

If scarring was present, the patient could fall into one or more of the categories listed above.

### Ethics

Ethics approval was obtained from the Research Ethics Committee of Stellenbosch University (ref. no. S18/09/181), and an application for a waiver of consent was agreed upon owing to the retrospective nature of the study.

### Statistical aspects

Descriptive statistics and χ² comparisons of proportional data were performed. A p-value of <0.05 in a two-tailed test of proportions was considered significant.

## Results

There were a total of 917 patients identified, and 268 (29.2%) were found to have presence of scarring (Table 1). The patients had a mean age of 60 years and were mostly (60%; n=553) males. Patients with scarring matched those without scarring with regard to their age (59 years v. 60 years) and ECOG status (2 v. 2). There was a higher percentage of males in patients with scarring present (67% v. 57%; p=0.004). Of the patients for whom a smoking history was documented, 611 were found to be smokers and 57 non-smokers. Smoking was associated with a higher likelihood of presence of pulmonary scarring, at 30.3% (n=189), compared with 19% (n=11) of non-smokers (p=0.043) demonstrating scarring.

The most common histological subtype of non-small-cell lung cancer was adenocarcinoma, with the majority of patients having stage IV disease. Small-cell cancer was identified in 126 patients with extensive disease being most prevalent in this group. The presence of pulmonary scarring in the same lobe as the primary tumour was not associated with a specific histological subtype of cancer.

The presence of scarring was found in 268 (29.2%) patients. Of these patients, 164 areas of pulmonary scarring was found in the same lobe as the primary tumour (Table 2). A total of 33 patients had diffuse fibrosis, which was included as patients with pulmonary scarring in the same lobe as the primary tumour. There were 153 patients with scarring in other lobes of the lung. Ninety-four patients had scarring present in 2 or more lobes (i.e. categories) that did not fall into the diffuse group.

Using a χ² analysis, in patients with lung cancer, if scarring was present it was more likely to be present in the same lobe as the primary tumour. This was compared with scarring in any other areas of the lung (n=197 v. n=153; p<0.001). Furthermore, if scarring was present in the same lung as the primary tumour it was more likely to be in the same lobe as the tumour (n=154 v. n=71; p=0.004).

## Discussion

We found a strong association between having scarring and developing lung cancer in the same anatomical location as the tumour. Almost one in three patients had scarring present, and scarring was more likely in the same lobe as the tumour, or having diffuse fibrosis. The histological subtype, performance and age were all comparable between the two groups. Males made up 60% of the cohort, which falls in with a much higher reported percentage of lung cancers in males.^[2]^ There was also male predominance in the scarring group compared with the non-scarring (67% v. 57%), which is similar to a previous study showing male predominance in patients with lung cancer with scarring at baseline.^[5]^ Scarring was also found to be more likely in patients with a history of smoking (70% v. 65%).

In previous studies, an association was found between the anatomical location of scarring and the presence of lung cancer, but these where small and mostly histological studies.^[8,10,12,16]^ The results of these studies showed presence of scarring in 4 - 20% of histological examinations of patients with lung cancer. Yu *et al*. ^[5]^ found that scarring was present in 15% of patients with lung cancer; our study found almost double this amount. This could be related to the high incidence of TB in our patient population in the Western Cape Province^[14]^ as TB is postulated to contribute to defective tissue repair and fibrogenesis even with optimal treatment.^[17]^ In a meta-analysis, TB was found to an independent risk factor to develop lung cancer even 20 years after exposure to pulmonary TB,^[18]^ and in a more recent study, ‘scarcinomas’ seemed to have a predilection to TB sites, and if present, the tumour was usually larger (3.5 cm v. 5.3 cm).^[19]^ Thus it is possible, given our observations, that post-tuberculosis lung fibrosis could increase the risk of lung cancer in our population. However, with the high prevalence of smoking found in our patients (67%), determining whether post-TB scarring was an independent risk factor for the development of ‘scarcinoma’ would require further investigation. Our study did not find a predilection to a specific histological subtype if scarring was present in the same lobe as the tumour. We postulate that because this was a radiological study, and identifying ‘scarcinoma’ is notoriously difficult based on radiology alone,^[18]^ probably not all of the tumours present in the area of scar were ‘scarcinomas’.

The proposed pathogenesis of scar carcinoma is thought to be that of atypical bronchiolar proliferation that becomes excessive during regeneration, and may predispose to malignant change.^[7,9,16]^

There is also evidence of hyperplasia and occasional malignant changes of the bronchiolar and alveolar epithelium in the margins of pulmonary infarctions, in areas of organising pneumonitis, diffuse fibrosis, scleroderma and rheumatoid lung.^[20]^

Another proposed pathogenic mechanism is that of blockage of lymphatic and venous drainage, with carcinogen pooling within the scar tissue.^[21]^ Given genetic variation^[13]^ and a lack of a relationship to smoking,^[8]^ ‘scarcinomas’ can potentially be seen as separate pathological entities as compared with non-scar carcinomas. ‘Scarcinomas’ traditionally behave differently to non-scar carcinomas. They can present with a negative chest radiograph, but with extra-pulmonary manifestations related to metastatic disease, and may indicate a poorer prognosis.^[22]^ These ‘scarcinomas’ were traditionally described as being small (<3 cm), often found in the periphery of the lung and in an area of existing scarring.^[6]^ This makes them difficult to detect on a regular chest X-ray (CXR) to the untrained eye. This entity should be considered for any patient who presents with scar tissue present on a CXR with a possibility of cancer (unexplained loss of weight or metastatic disease with an unknown primary). When suspecting the diagnosis of ‘scarcinomas’, comparing previous radiology is essential, to look for enlargement of scar tissue or new nodules in the area of scarring.^[19]^

### Study limitations

This study has some limitations owing to it being retrospective, and the fact that only patients with a confirmed histological diagnosis and staging CT scan were included. Another limitation is that ‘scarcinoma’, as a separate entity, was challenged in the 1980s, as most cancers have the ability to produce fibrosis. Therefore proving whether fibrosis was present before the carcinoma is challenging. The presence of dense hyaline scarring in the centre of many primary peripheral lung carcinomas led to the recognition of this entity. However, cases thought to be ‘scarcinoma’ were reviewed in three histological studies where high levels of type III collagen were found. This suggests an immature and ongoing fibrotic process, and would be in keeping with a desmoplastic reaction resulting from the host response to the carcinoma, rather than old fibrosis preceding the carcinoma.^[23–25]^ Therefore determining if scarring of lung tissue was a desmoplastic reaction or that of original scar tissue preceding the tumour is difficult with radiology alone. Histology of samples to look at collagen type and further prospective studies may be of benefit to strengthen the aim of the study. 

A history of past TB would have been beneficial in this study, with our high TB burden. Being able to say that scarring alone, in the setting of previous TB, would be an independent risk factor is not possible given the methodology of this study. Lastly, a specialist radiologist was not present to review the CT scans that were reviewed by the authors, but the radiological reports were accessed, compared with our findings and taken into account. The authors were also not blinded to each other’s opinion, and this could result in potential bias.

## Conclusion

In conclusion, we found a strong association between the anatomical location of scar tissue and the presence of lung cancer.

## Figures and Tables

**Table:** Table 1. Demographics, lung cancer type, staging and performance status of all patients (n=917)

	Scarring present (N=268), n (%)^*^	Scarring absent (N=649), n (%)^*^
Age (years), mean (SD)	59	60.5
Gender		
Male	182 (67)	372 (57)
Smoking status		
Smoker	189 (70.5)	422 (65)
Lung cancer type		
Adenocarcinoma	119 (44.4)	296 (45.6)
Squamous cell	88 (32.8)	156 (24)
Poorly differentiated	27 (10)	73 (11.2)
SCLC	32 (11.9)	94 (14.6)
Other	2 (0.7)	30 (4.6)
NSCLC staging (N=791)^†^		
I	4 (1.7)	14 (2.6)
II	5 (2.1)	17 (3.2)
III	68 (29.6)	143 (26.7)
IV	153 (66.5)	362 (67.5)
SCLC staging (N=126)		
Limited	4 (12.5)	17 (18.1)
Extensive	28(87.5)	77 (81.9)
ECOG performance status		
1 - 2	158 (59)	399 (61.5)
3 - 4	81 (30.2)	155 (23.9)

SD = standard deviation

SCLC = small-cell lung cancer

NSCLC = non-small-cell lung cancer

ECOG = Eastern Cooperative Oncology Group

^*^ Unless otherwise specified

^†^ Of the 791 patients, 230 had scarring and 536 had no scarring

**Table:** Table 2. Summary of the presence and distribution of scarring (in patients (n=268) and per anatomical site (n=377))

	Area of scarring present (n=377), n(%)	Patients (n=268), n(%)
Same lobe as cancer ^*^	164 (44)	-
Only present in the same lobe	-	82 (31)
Present in same lobe and in DLSL	-	28 (10)
Present in same lobe and in CL	-	39 (15)
Present in same lobe, DLSL and CL	-	15 (6)
Different lobe, same lung^†^	71 (19)	-
Only present in DLSL	-	16 (6)
Present in DLSL and in CL	-	12 (4)
Contra-lateral lung^‡^	109 (29)	-
Only present in CL	-	43 (16)
Diffuse fibrosis	33 (8)	33 (12)
Fibrocystic changes	15 (46)	-
IPF	10 (30)	-
Bronchiectasis	4 (12)	-
Other	4 (12)	-

DLSL = different lobe, same lung

CL = contralateral lung

IPF = idiopathic pulmonary fibrosis

^*^ Patients where scarring is present in the same lobe as the cancer

^†^ Patients where scarring is present in the same lung as the cancer but in a different lobe (DLSL)

^‡^ Patients where scarring is present in the contralateral lung (CL)
